# High mobility group box 1 antagonist limits metastatic seeding in the lungs via reduction of cell–cell adhesion

**DOI:** 10.18632/oncotarget.16188

**Published:** 2017-03-14

**Authors:** Adi Karsch-Bluman, Benzion Amoyav, Nethanel Friedman, Hila Shoval, Ouri Schwob, Ezra Ella, Ori Wald, Ofra Benny

**Affiliations:** ^1^ The Institute for Drug Research, The School of Pharmacy, Faculty of Medicine, The Hebrew University of Jerusalem, Jerusalem, Israel; ^2^ The Goldyne Savad Institute of Gene Therapy, Hadassah-Hebrew University Medical Center, Jerusalem, Israel; ^3^ Division of General Thoracic Surgery, Michael E. DeBakey Department of General Surgery, Baylor College of Medicine, Houston, TX, USA

**Keywords:** HMGB1, ICAM1, LLC, Carbenoxolone, metastasis

## Abstract

Metastatic spread is the leading cause for cancer-related mortality, with the lungs being a major site for metastatic seeding. Available therapies for patients with metastatic disease are extremely limited. Therefore, there is a desperate need for new strategies to prevent or limit metastatic dissemination and treat existing metastases. The metastatic cascade is highly complex and is affected by multiple factors related to both tumor cells themselves and the microenvironment in the future site of metastasis. We hypothesized that modifying the lung microenvironment by blocking central ubiquitous signals may affect metastatic seeding in the lungs. Given the high basal levels of the Receptor for Advanced Glycation End products (RAGE) in the pulmonary tissue, and its pro-inflammatory properties, we investigated the consequences of interfering with its ligand; High Mobility Group Box 1 (HMGB1). To this end, we tested the effect of Carbenoxolone, an HMGB1 antagonist, on primary tumor growth and metastatic progression in several murine tumor models. We show that antagonizing HMGB1 prevents the adhesion and colonization of cancer cells in the lungs through the reduction of their adhesion and cell–cell interaction both *in vitro* and *in vivo*. We demonstrated that these activities are mediated by downregulation of the adhesion molecule Intercellular Adhesion Molecule 1 (ICAM1) and ultimately result in reduced metastatic burden. Carbenoxolone decreases significantly lung metastases formation and can be used potentially as prophylactic therapy for metastatic diseases.

## INTRODUCTION

The lung, the second most targeted organ for metastases formation, is a massively perfused tissue that possess physiological and environmental traits which accommodate growth of seeded cancer cells post dissemination [1, 2]. While there is a wide panel of both chemical and biological therapies, metastatic cancers remain mostly incurable and none of the clinically available drugs have yet to be found effective as preventative anti-metastatic treatment.

Metastases formation in the lung often leads to respiratory failure and consequently to death. Therefore, diminishing the metastatic potential of cancer cells to the lung, would have an enormous effect on patients diagnosed with cancer. Aiming at identifying potential anti-metastatic targets in the lung, we focused on the natural biological pulmonary niche. The natural microenvironment of the pulmonary tissue plays an important role in metastatic cell colonization and in the progression of the disease. Previous studies show a strong link between metastases and inflammation [3–5], a process in which the Receptor for Advanced Glycation End products (RAGE) plays a major role [6]. The pulmonary tissue in known to express exceptionally high basal levels of RAGE compared with other healthy tissues [7]. One of the identified ligands of RAGE is the High Mobility Group Box1 (HMGB1) protein. HMGB1 is both a nuclear factor and a secreted protein. HMGB1 is expressed in all mammalian cells and is overexpressed in various types of cancer cells [8–11]. Though normally bound tightly to chromatin, HMGB1 can be secreted from cells undergoing necrosis. It binds extracellularly with high affinity to RAGE and is a potent mediator of both inflammation and angiogenesis [12]. Recent studies have identified Carbenoxolone to be an antagonist of endotoxin-induced secretion of HMGB1. Carbenoxolone is a drug chemically derived from Glycyrrhizine, a traditional remedy for inflammatory ailments made of Gancao (Radix Glycyrrhizae; Licorice) extraction. Carbenoxolone is made by the replacement of the glucuronic acid with succinic acid. It is a licensed drug in the UK, prescribed for both esophageal ulceration and inflammation [13].

Our hypothesis, given the anti-inflammatory trait of Carbenoxolone and the known role of inflammation in cancer, is that RAGE signal may be a central player in controlling metastases, especially in the lung. We therefore suggest that biochemical downregulation of RAGE's pathway would lead to the reduction in these metastatic processes. In light of Carbenoxolone's previously demonstrated reduction of HMGB1 release in cells under stress [13], and since it is already in use for other indications in traditional medicine, we aim to study the effect of this compound on specific cellular steps in the formation of metastatic lesions in the lung.

In this study, we performed a panel of cell based assays aimed at identifying the effect of Carbenoxolone on specific steps of tumor growth and metastases. We chose Lewis Lung Carcinoma (LLC) murine cell line because of its highly metastatic potential and the possibility to evaluate both primary and secondary growth in the relevant microenvironment of the lung. In order to study both tumor progression and spread of metastasis, we performed four different *in vivo* models. The *in vivo* models applied were two primary tumor models: subcutaneous and orthotopic, and two metastatic-relevant models: cell pulmonary colonization and tumor resection model for spontaneous cancer cell spread. Our findings have established that the primary anti-cancer activity of Carbenoxolone is on the metastatic process rather than on the localized growth of the primary tumor. We show that the drug impairs lung carcinoma cells from forming colonies, a process associated with reduction in the cell–cell adhesion molecule, intercellular adhesion molecule1 (ICAM1), and hinders their ability to adhere to the Extra Cellular Matrix (ECM).

There is great clinical promise in the use of a drug that is already available for other indications, to prevent the spread of tumors, the leading cause of death in many cancers. Understanding the underlining cellular mechanism may allow us to design an improved formulation with regard to drug pharmacokinetics and frequency of administration. Since metastatic cancer in the lung remains incurable and, most significantly, none of the offered treatments are used as prophylactic therapy for metastases, we suggest, based on our data, to further investigate the potential of Carbenoxolone in the prevention of metastases following primary tumor diagnosis.

## RESULTS

### Functional consequences of carbenoxolone

#### Carbenoxolone prevents HMGB1 secretion and affects cell growth and mobility

**Figure 1:**
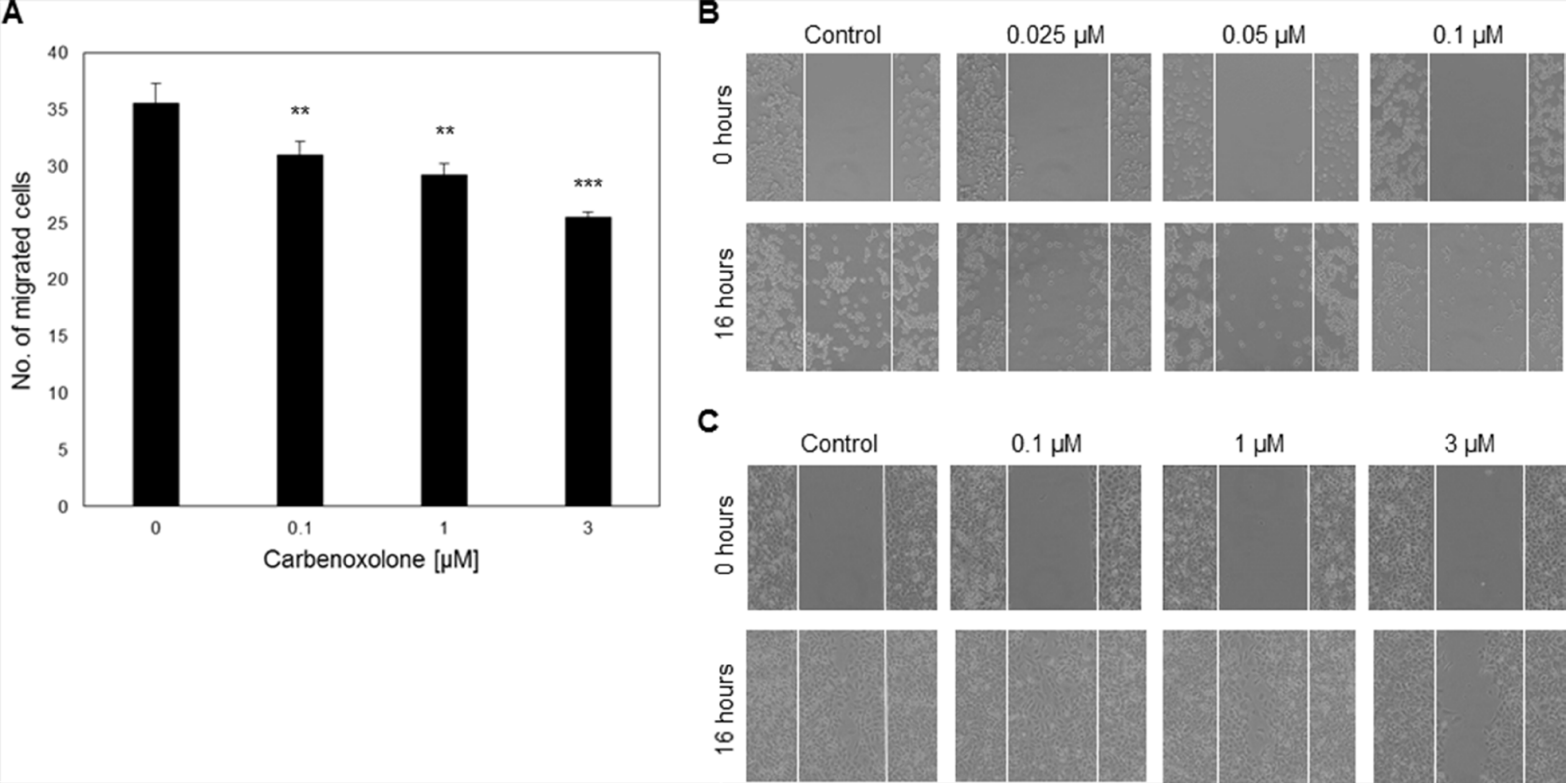
Carbenoxolone as an HMGB1 blocker and cell migration inhibitor (**A**) Transwell assay of LLC cells after 21 hours of incubation with ranging concentrations of Carbenoxolone, presenting dose response migration. *n* = 6. (**B**–**C**) Scratch assays at t0 and after 16 hours of incubation with ranging concentrations of Carbenoxolone. White thin lines present the scratch area. (B) LLC cells were incubated with lower concentrations than (C) MDA-MB-231 cells. At t0 inserts were removed and 16 hours later pictures were taken of both LLC and MDA-MB-231. *n* = 3. **p ≤ 0.01, ***p ≤ 0.001.

We confirmed that Carbenoxolone blocks the secretion of HMGB1 from activated cells by performing an LPS macrophage activation assay over 24 hours as previously published [14]. Level of HMGB1 in lipopolysaccharide (LPS) activated macrophages was assessed using immunoblot analysis. Results show that Carbenoxolone inhibits LPS-induced HMGB1 secretion, while the intracellular HMGB1 level remains high in all tested concentrations of 10–100 μM (Supplementary Figure 1). Data was also confirmed with cellular staining of HMGB1, demonstrating nuclear localization (data not shown). Next, we wanted to assess the effect of Carbenoxolone on cell functions related to tumor progression and metastases. Therefore, we measured the result of Carbenoxolone on cell proliferation and viability in murine fibroblasts (NIH/3T3), human melanoma cancer cell line (A-375) and LLC cells. As shown in Supplementary Figure 2, Carbenoxolone demonstrated a minor effect on the proliferation of LLC and the proliferation of A-375 and NIH/3T3 was inhibited in up to 30% and 46% with 10 μM, respectively. Since the activity of inhibiting cell viability in LLC cells was relatively modest, we followed up by assessing whether cell mobility is affected more dramatically by the drug. First, the effect of Carbenoxolone on cell migration was studied using both scratch and transwell assays (Figure 1, Supplementary Figure 3). In the scratch assay, initially, both MDA-MB-231 human breast cancer and LLC cell lines were treated with equal Carbenoxolone concentrations (0.1–3 μM). However, LLC presented early detachment, therefore, the exposure of LLC to the drug was decreased to 0.025, 0.5 and 0.1 μM of Carbenoxolone. MDA-MB-231 cells reached complete coverage in three of the four samples after 16 hours of incubation. In both cell lines, the capacity of cells to migrate was diminished compared with the untreated cells. Transwell assay performed for 21 hours revealed that Carbenoxolone significantly decreased cell migration in LLC cells in a dose dependent manner, showing 13%, 18% and 28% decrease with 0.1 μM and 1 μM and 3 μM respectively.

### Exposure to carbenoxolone increases susceptibility of cancer cells to anoikis

**Figure 2:**
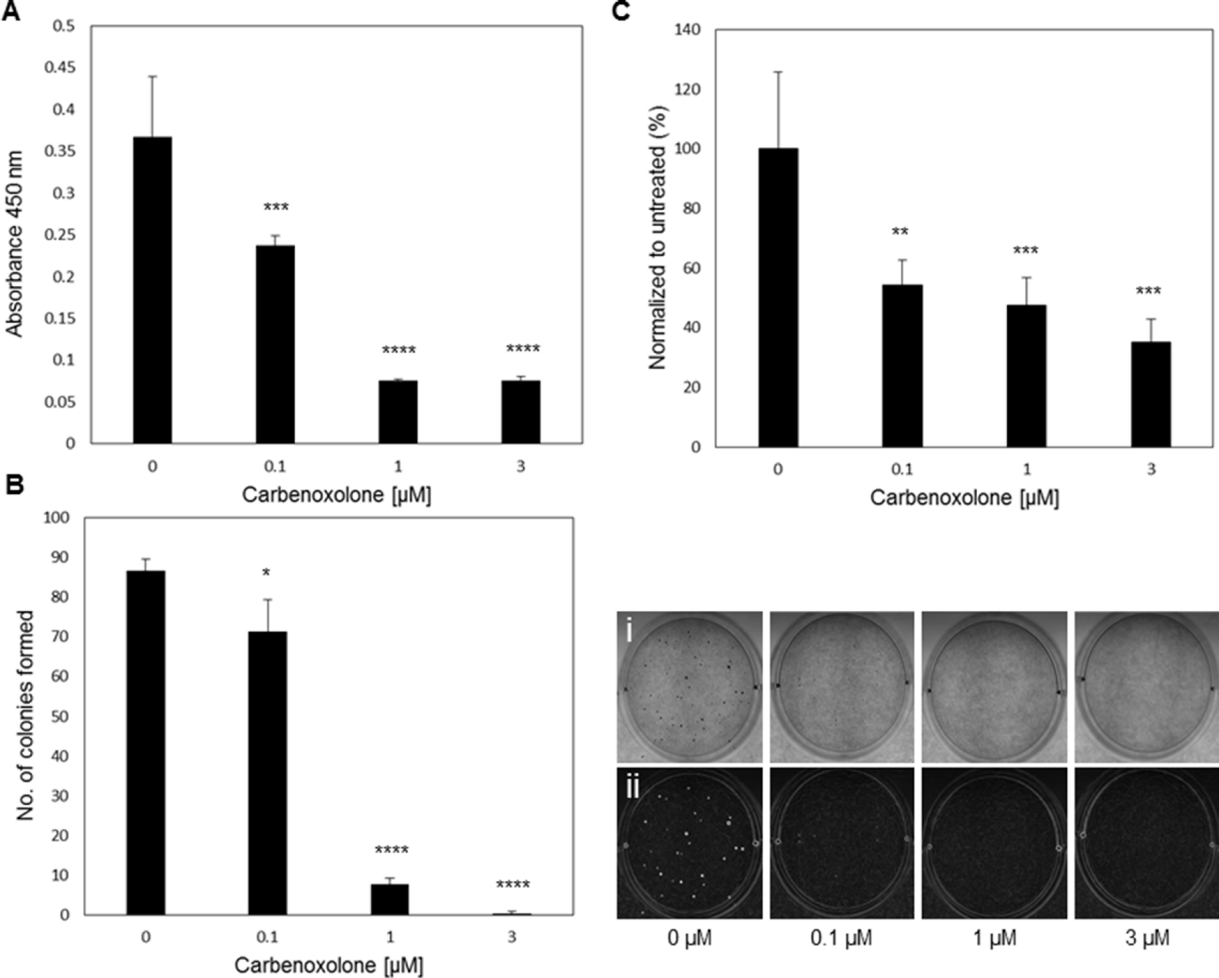
Carbenoxolone increases cells susceptibility to anoikis, and reduces adhesion and colony formation (**A**) Anoikis assay using p-HEMA coated plates on LLC after 72 hours of incubation with ranging concentrations of Carbenoxolone. *n* = 6. (**B**) Soft agar colony formation assay after 12 days of LLC cells under Carbenoxolone. Cells were stained with Giemsa, counted (left) and photographed (right) (i-eye. ii- contrast). *n* = 4. (**C**) Adhesion assay on collagen coated surface after 1 hour of Carbenoxolone incubation, normalized to uncoated surface. *n* = 6. *p ≤ 0.05, **p ≤ 0.01, ***p ≤ 0.001, ****p ≤ 0.0001.

Since cell survival in blood circulation is a key factor in the metastatic cascade, we evaluated the activity of Carbenoxolone in modifying non-adherent cell survival. In this assay, we tested the level of resistance to apoptosis of detached LLC cells treated with Carbenoxolone for 72 hours, by detecting cell viability on a non-adherent surface using WST8 (Figure 2A, Supplementary Figure 4). Images show that control cells had higher survival rates than treated cells. Figure 2A indicates an increased susceptibility to cell death as a result of Carbenoxolone treatment, presented by lower level of cell viability. While 0.1 μM significantly decreased viability in 35%, 1 and 3 μM induced significantly higher cell death with a decrease of 79% in cell viability, compared with the untreated cells.

### Carbenoxolone decreases cell adhesion to ECM and colony formation

To investigate the potential effect of low dose Carbenoxolone on cell adhesion, a crucial step in colonization and cancer metastasis, we performed *in vitro* assays examining the level of adherence of LLC cells under Carbenoxolone treatment. Plates coated with varying extracellular matrix components: fibronectin, collagen (I), laminin, elastin and gelatin, with an uncoated plate as control, were seeded with LLC cells under Carbenoxolone treatment (Figure 2, Supplementary Figure 5). Data show that the effect of Carbenoxolone treatment on the adherence of cells was most prominent in the collagen coated plates (Figure 2C). LLC cells seeded on a collagen coated surface presented a 46% reduction of adhesion when treated with 0.1 μM Carbenoxolone, compared with no effect in the uncoated plate, and up to 53–65% reduction, respectively, after 1 hour of incubation with 1 and 3 μM Carbenoxolone. In the uncoated control plate, a decrease of 20–31% in 1 and 3 μM Carbenoxolone, respectively, was detected. In laminin and gelatin coated surfaces there were moderate effects on LLC attachment in response to Carbenoxolone treatments. Adhesion of LLC cells to fibronectin, elastin and gelatin coatings was less affected than to collagen coated surface.

Beyond cell adhesion, the ability of cancer cells to form colonies in soft agar matrix correlates with their metastatic potential and tumorigenicity [15]. The capacity of LLC cells to form colonies was determined by the soft agar assay over 12 days. Figure 2B shows a clear dose-dependent effect of Carbenoxolone on the ability of cells to form colonies. The number of colonies formed under 0.1 μM incubation was 18% less than that of the untreated group, but the higher concentrations provided a more significant effect with a decrease of 89% and 96% for 1 and 3 μM, respectively.

### Carbenoxolone decreases microvessel formation and HMGB1 levels in tumor tissues without affecting their volume

**Figure 3:**
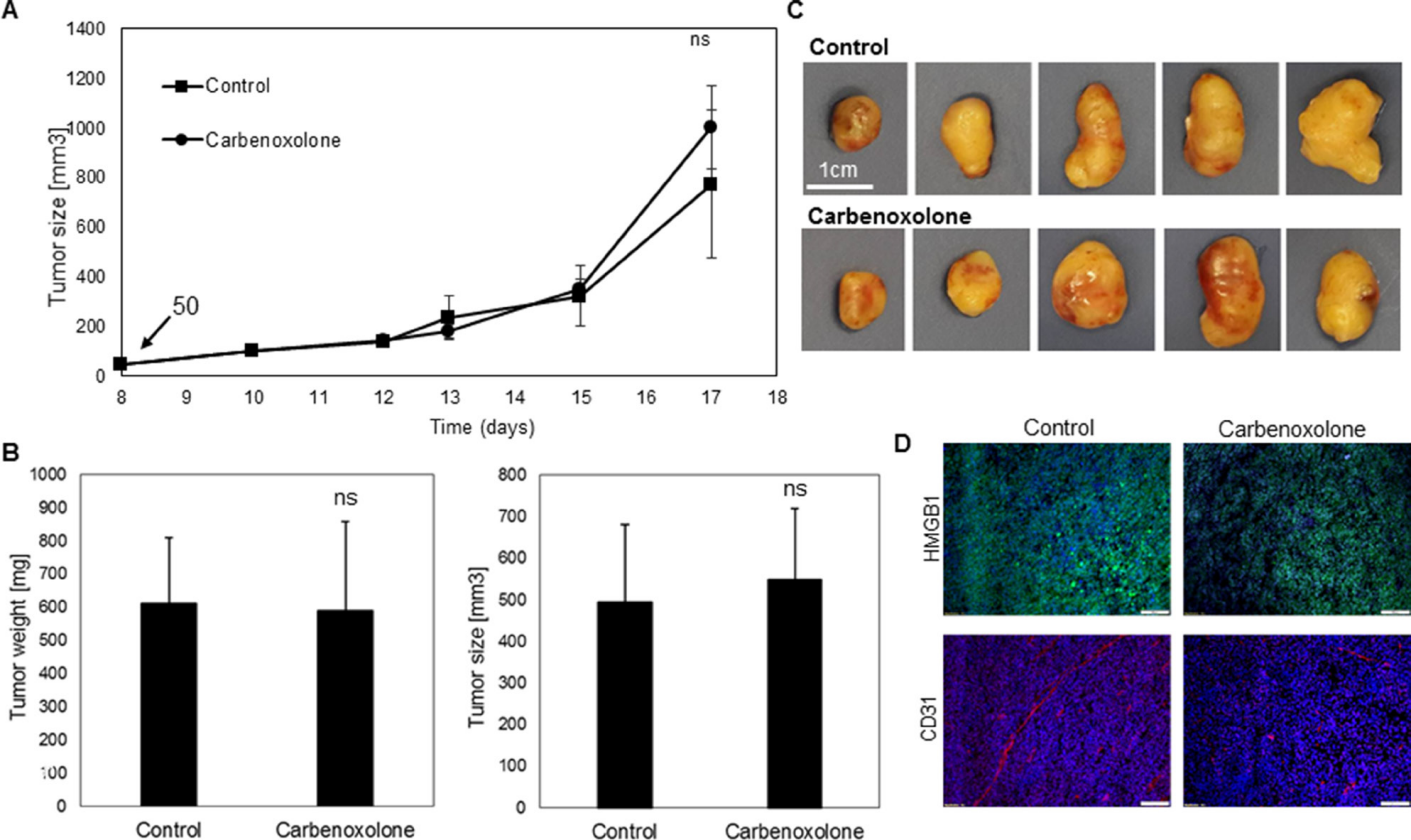
Effect of Carbenoxolone on s.c. tumors growth and HMGB1 and CD31 levels (**A**) Tumors started developing 8 days post LLC cells s.c. injections in C57BL/6 mice. Treatment administered on the 10^th^ day- 50 mg/kg i.p., with PBS as control, q.o.d. Tumor volume was measured using a caliper. End point was on the 17^th^ day and tumors were harvested. (**B**, **C**) Weight and volume of tumors post resection. (**D**) Representative figures of tumor sections stained for HMGB1 and CD31. *n* = 8. ns = non significant.

To investigate whether the *in vitro* effects of Carbenoxolone are also reflected *in vivo*, a subcutaneous tumor experiment was performed using LLC cells in C57BL/6J mice. Treatment (50 mg/kg) was initiated when the tumor volume reached 100 mm^3^, presenting no statistically significant change in volume over the course of treatment (Figure 3). At the end point, after 17 days of treatment, no significant differences of tumor size and weight between the treated and untreated mice were detected, with average weights of 619 mg or 590 mg per tumor, and volumes of 494 mm^3^ or 549 mm^3^ of the control and treated groups respectively (Figure 3A–3C).

Based on the previous indications that Carbenoxolone can act as an HMGB1 antagonist, levels of HMGB1 were analyzed in tumor tissues by immunohistology, showing lower HMGB1 levels in the treated tumors compared with the untreated group (Figure 3D). Moreover, an immunoblotting experiment was conducted to quantify HMGB1 level in the tumors of both treated and untreated groups. Results in Supplementary Figure 6, further support Carbenoxolone's inhibiting effect on the expression of HMGB1. In addition, the vascularity state of tumors was analyzed using specific CD31 staining. Lower microvessel formation was detected in treated tumors compared with the control group, despite similar volume. The lack of difference in the size of the primary tumors suggests that while there is anti-angiogenic activity, as seen by the reduction in microvessels formation in the Carbenoxolone treated group, there may be additional processes that compensate and prevent the shrinkage of the tumor.

### Carbenoxolone has no effect on primary tumor growth in the orthotopic lung model

**Figure 4:**
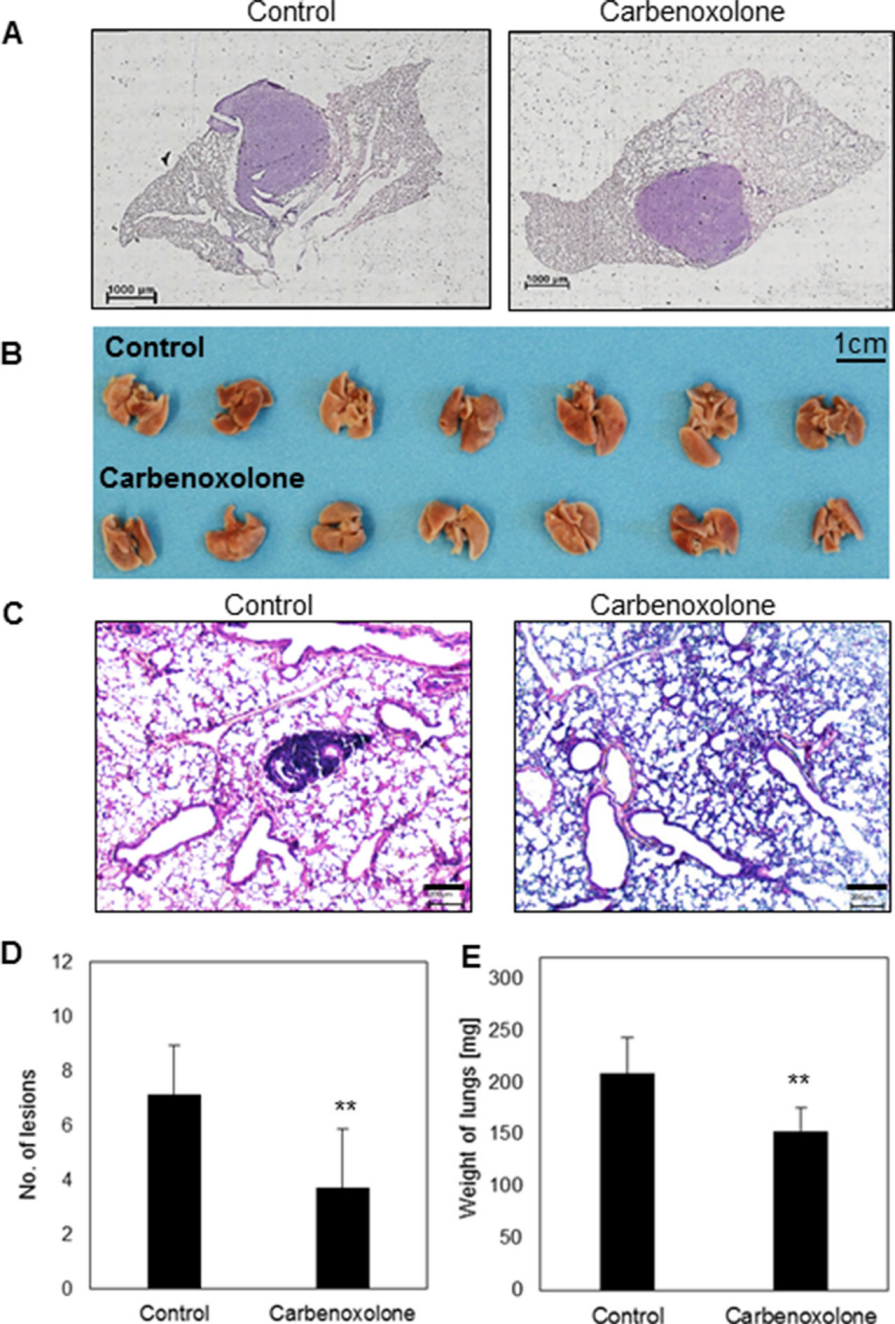
Effect of Carbenoxolone in orthotopic primary growth and tail vein injection (**A**) An orthotopic model- representative histologic sections of LLC tumor in the left lung of C57BL/6 mice untreated (left) and treated (right) with 10 mg/Kg/d of Carbenoxolone for 17 days. Drug was administered 3 days after cancer cells were injected into the lung. (**B**) Tail vein systemic injections on C57BL/6 mice. Mice were pre-treated i.p. with 50 mg/kg Carbenoxolone q.o.d., (PBS as control). On the 5^th^ day all mice were injected IV with LLC cells (5 × 10^6^). On the 8^th^ day dose was reduced to 40 mg/kg. End point was 21 days after treatment started due to the death of a mouse from the control group, and lungs resected (**C**) Representative histologic sections of lungs of control (left) and Carbenoxolone treated (right) mice after tail vein experiment post H&E staining (4×). (**D**) Number of lesions found in lungs of untreated mice compared with lesions found in lungs of mice treated with Carbenoxolone, *p* = 0.006. (**E**) Weight of Lungs of untreated mice compared with that of mice under Carbenoxolone treatment, *p* = 0.004. *n* = 8. **p ≤ 0.01

In order to study LLC growth in its organ-specific microenvironment, we used an orthotopic model which allows the tumor to grow in a single site in the lung, similar to the clinical phenotypic growth. C57BL/6J mice were injected with LLC cells directly into the lungs [16]. Treatment was administered intraperitoneally (i.p.), 10 mg/kg per day, 3 days after LLC cells were injected into the lungs, over a course of 14 days. Histological sections of the left lung (Figure 4A) post Hematoxylin/Eosin (H&E) staining, demonstrated no differences in tumor volume as a result of Carbenoxolone treatment.

### Carbenoxolone diminishes both number and size of lesions in the lungs

To investigate the effect of Carbenoxolone on colonization of carcinoma cells in the lung, we performed tail vein injections of LLC in C57BL/6J mice. 21 days after cell injections the experiment was terminated, mouse lungs were removed and H&E staining of histological sections were performed. Staining showed that Carbenoxolone reduces both the number and size of lesions in the lungs (Figure 4B–4E). To quantify the results, we used a scoring system based on lesion size as follows: small (< 50 μM), medium (50–250 μM) and large lesions were counted in each tissue section (250 μM <). Mean numbers on lesions found on lungs of control and treated groups respectively were: large 2 and 1; medium 4 and 2; small 6 and 2 (average of 7 slides per group). Results are in correlation with the significant difference of lung weight between treated and untreated groups, yielding 26% weight reduction in mice treated with Carbenoxolone.

### Carbenoxolone reduced metastases burden in lungs

**Figure 5:**
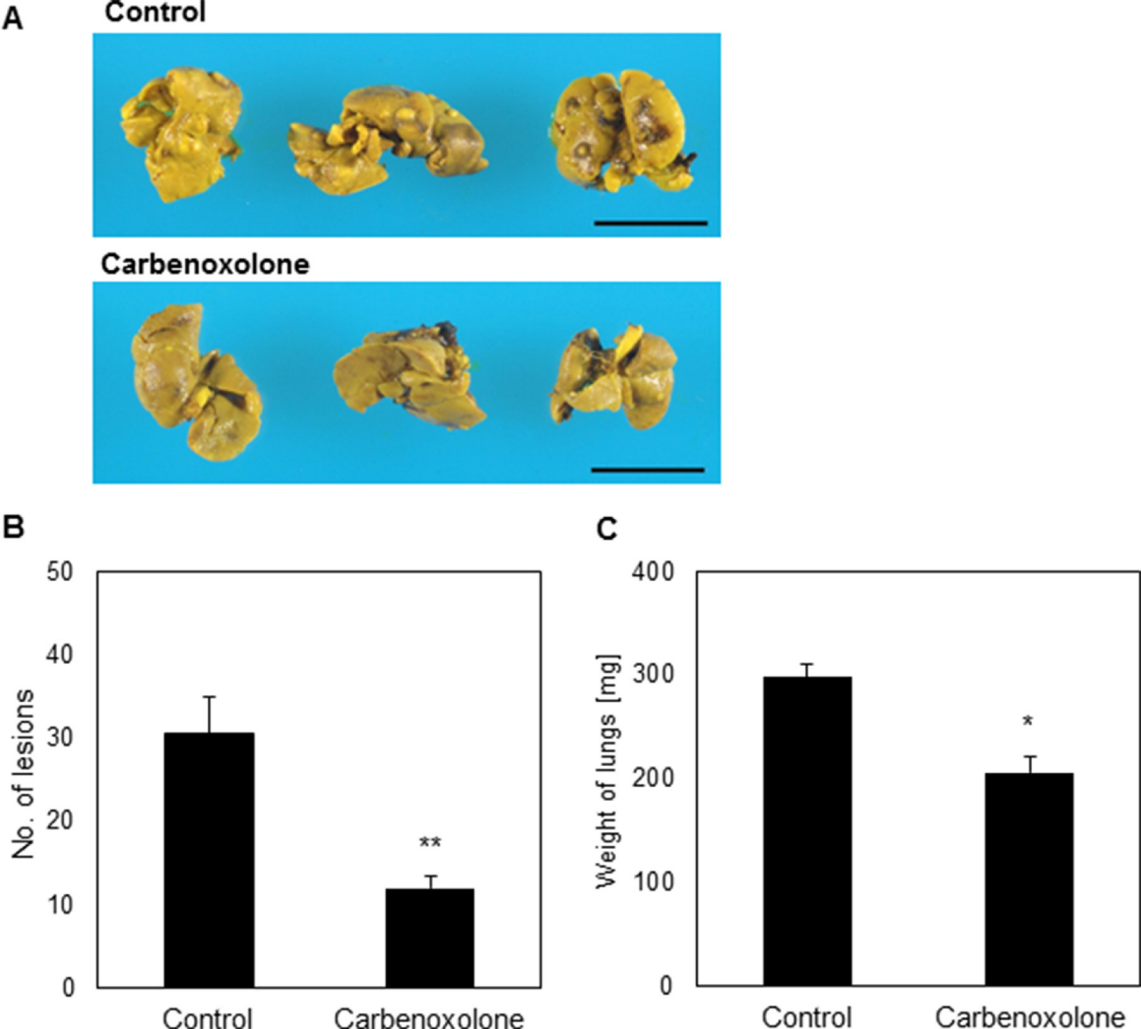
Effect of Carbenoxolone in tumor resection model (**A**) C57BL/6 mice were inoculated s.c. with 1.5 × 10^6^ LLC cells. A week prior to injection of cells, treatment of CAR 50 mg/kg i.p. q.o.d. was administered. When tumors reached volume of ~1000 mm^3^ (day 10) tumors were resected surgically and mice were monitored for an additional 21 days to allow metastases to develop. Lung resected and pictures were taken. (**B**) Number of lesions found in lungs of untreated mice compared with that found in mice treated with Carbenoxolone, p = 0.002. (**C**) Weight of lungs of untreated mice compared with lungs of mice under Carbenoxolone treatment *p* ≤ 0.05. *n* = 7. *p ≤ 0.05, **p ≤ 0.01

Spontaneous spread of cells from the primary site represents a more clinically relevant model for metastasis formation, as compared to tail vein injection. Therefore, we used the tumor resection model which mimics the clinical onset of metastatic formation in the lung; a cascade involving tumor dissemination, circulation, adhesion, intravasation and colonization in distant organs. Lungs resected from untreated mice were widely speckled with lesions, as opposed to lungs of mice that were treated with Carbenoxolone (Figure 5). The number of lesions on lungs of treated mice was counted and found significantly lower compared with the untreated group. The average numbers of lesions found in the lungs were 12 and 31 of treated and untreated mice respectively. Average weight of lungs resected from untreated mice was 31% higher than the treated group, which can be attributed to the higher number of metastasis and to the development of edema.

### Carbenoxolone reduces levels of HMGB1 and ICAM1 in cells

**Figure 6:**
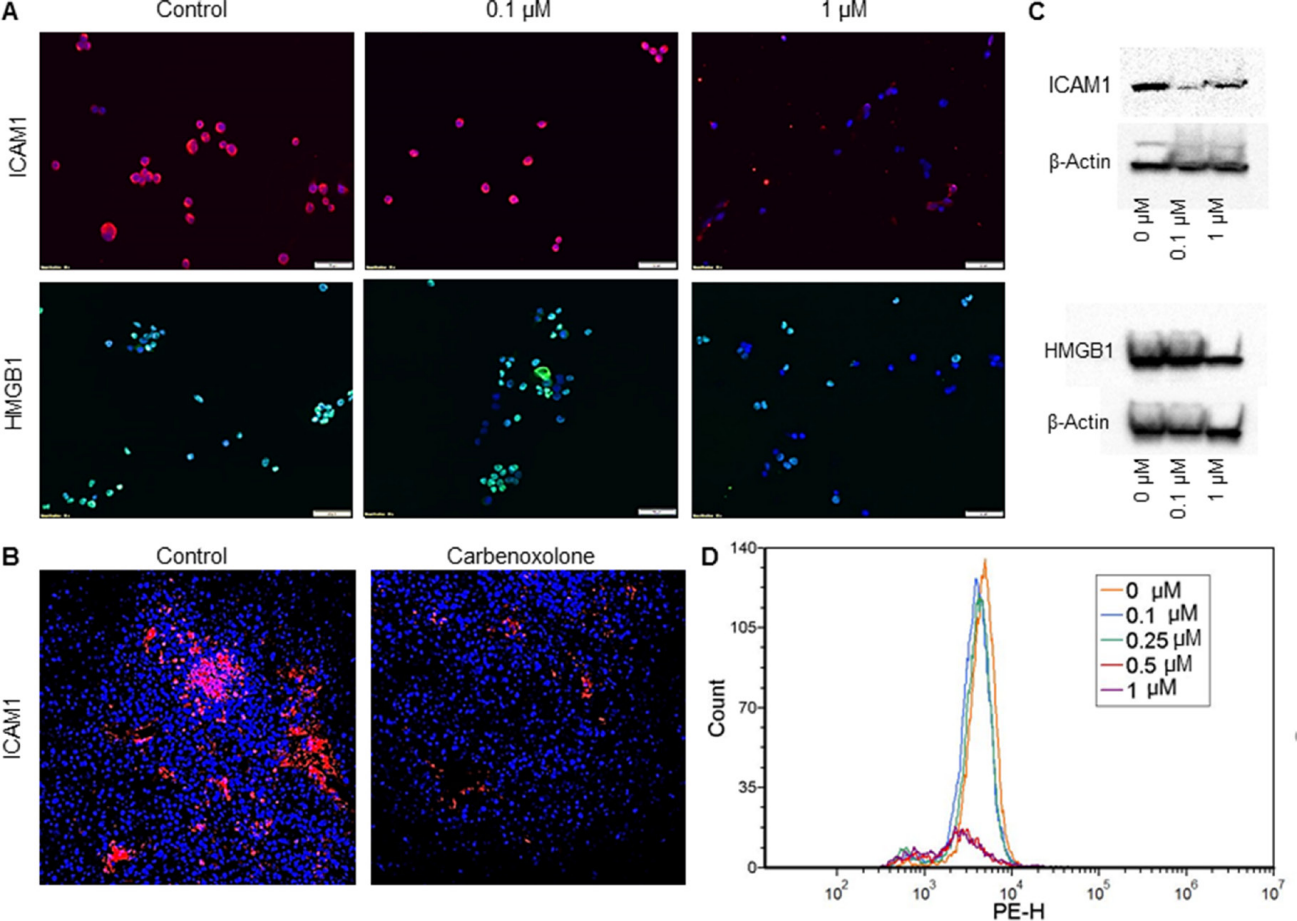
Effect of Carbenoxolone on HMGB1 and ICAM1 expression levels (**A**) Immunofluorescence staining of LLC cells after 1 hour incubation with Carbenoxolone. 50K cells/well were seeded in a 24 well culture dish and incubated overnight. Cells were stained for nuclei with DAPI (blue) and either ICAM1 (Cy3-red) or HMGB1 (Alexa flour 488 -green). (**B**) Tumor sections of Carbenoxolone treated mice stained for ICAM1 and DAPI. *n* = 8. (**C**) Western blot analysis of ICAM1 (top) and HMGB1 (bottom) expression in LLC cells post 1 hour incubation with Carbenoxolone. Blots are cropped, full blots can be found in Supplementary Figure 7 (**D**) Flow cytometry analysis of LLC cells incubated with for 1 hour. Fluorescent detection of ICAM1 was measured using Cy5. Duplicates of all samples provided the same effect.

Following our data of the *in vitro* colony formation assay and our *in vivo* results, we further elucidated the mechanisms involved in LLC colonization in the lung. For this purpose, we assessed the level of ICAM1 as a key cell–cell adhesion molecule. Cells treated with Carbenoxolone show reduction of both HMGB1 and ICAM1 protein levels in immunostaining, while 1 μM treatment provided considerably lower levels of ICAM1 (Figure 6). In addition, western blot analysis of LLC cells treated with Carbenoxolone showed an extensive decrease in the expression of ICAM1, and a detectable lower level of HMGB1 after incubation with the drug (Figure 6C). These results are further supported by flow cytometry analysis, demonstrating a concentration-dependent effect of Carbenoxolone on ICAM1 expression in LLC cells, with 0.5 and 1 μM providing a clear decrease in ICAM1 expression (Figure 6D). Based on these findings, we fluorescently stained the tumor section of our first subcutaneous model (as previously detailed) with anti-ICAM1 antibody (Figure 3, Figure 6B). Results show clearly that the treated mice presented far lower expression of ICAM1 in their tumors compared with the tumors of the untreated control group. These results suggest that the effect of Carbenoxolone on metastasis is regulated through attenuating the expression of ICAM1.

## DISCUSSION

The growing death rate worldwide as a result of highly metastatic cancers [17, 18], emphasizes the unmet need for therapeutic agents that can inhibit the uncontrolled growth of cancer cells and that can block their metastatic activity. Cell mobility, gained by a process called ‘Epithelial–Mesenchymal Transition’, enhances the potential of cells to penetrate and intravasate into the blood stream. Cells that survive the circulation, adhere to tissue of distant organs, and subsequently colonize to form micrometastases in their new microenvironment [19, 20]. It is well established that tissue microenvironment changes in cancer and the organ niche play an important role in the prosperity of cancer cells. We hypothesize that compounds that can target dominant signals of a specific organ niche, may modify the capacity of tumor cells to form metastasis in this organ. For the purpose of identifying compounds that can potentially affect the cascade of the formation of metastatic lesions in the lung, we followed the natural physiology of the lung along with promising leads of potential activity.

The Licorice root (Glycyrrhizae radix), has been in use for many years as a traditional remedy, mostly in China, for treating different ailments- especially inflammatory diseases. Glycyrrhizin is the main active component extracted from licorice roots and was proven to have anti-viral [21] and anti-inflammatory [22] traits. Studies comparing anti-cancer effects of Glycyrrhizin and its aglycon, Glycyrrhetinic acid (GA), in proliferation assays with murine melanoma cell line, found GA to be substantially more potent than its origin substance [23]. GA also presented anti-proliferative effects in leukemia [24] and human ovarian cancer cell lines [25], and was shown to target prostate cancer cells via an anti-inflammatory pathway through downregulation of HMGB1, IL-6 and IL-8 [26]. However, the compound has poor water solubility [27], thus presenting a pharmacological disadvantage. A water soluble derivative of Glycyrrhizin is Carbenoxolone [27]: a drug-like compound with low molecular weight and high solubility in water. Carbenoxolone is known for its anti-inflammatory activity, and was approved in the UK for treating gastric ulcers, due to the drug's low toxicity [13]. The primary anti-cancer properties associated with Carbenoxolone (demonstrated in different preclinical studies in cancers such as leukemia [28], thyroid [29] and mammary [30]), attributed most of their effects to the drug's mediation of connexin43 on gap junctions. Carbenoxolone was also shown to reduce inflammation in lungs [31] and airways, possibly by keeping lower levels of IL-4, IL-5 and eosinophils in the broncho-alveolar lavage [32]. However, no studies were performed to identify the direct activity of Carbenoxolone on metastases formation in the lung.

In light of previous indications, suggesting potential anti-tumor activity, and given that inflammation plays a key role in lung cancer pathogenesis [33], we aimed to investigate whether there are direct effects of the drug on the formation of metastasis in the lung, and attempted to determine the exact stages in the metastatic cascade that are involved. As stated previously, the lung tissue expresses high basal levels of RAGE receptor, unlike most healthy adult tissues [7]. Additionally, Carbenoxolone was found to be an antagonist of the cytokine activity of RAGE ligand, HMGB1 protein [13], a potent mediator of two cancer promoting processes; angiogenesis [13, 34] and inflammation [13, 34, 35].

After confirming that Carbenoxolone indeed inhibits the release of HMGB1, using the LPS activated macrophages assay (Supplementary Figure 1), we applied a systemic approach in which we investigated the effect of Carbenoxolone on several cell lines, using a variety of classical *in vitro* assays.

The anti-cancer activity of Carbenoxolone was examined using several cellular assays. Since primary lung tumors have the ability to metastasize to secondary sites in the pulmonary tissue or to other distant organs [36], we chose to work with LLC cell line. LLC is a highly metastatic cell model enabling the investigation of the role of the pulmonary specific tumor microenvironment, using an orthotopic model.

We sought to detect the activity on cell proliferation and migration, two important stages in the tumor progression process. Previously published data presented A-375 [37] and MDA-MB-231 [38], to have a concentration-dependent decrease in cell proliferation as a result of incubation with Licorice extracts. In an attempt to isolate the possible effect of Carbenoxolone on the tumor progression, we studied the proliferation of different kinds of cells. While both A-375 and NIH/3T3 presented a significant inhibition of 30% and 46%, respectively, with 10 μM Carbenoxolone, LLC cells demonstrated no clear effect on proliferation (Supplementary Figure 2). Our results support previous experiments that presented a lack of response in human prostate cell lines which were incubated with considerably higher concentrations of Carbenoxolone over 7 days [39]. Data suggest that rather than affecting proliferation, the main mechanism through which the drug acts in these cells is through targeting other metastatic cellular processes related to cell migration and invasion.

Based on the above, we further assessed cell mobility and invasion. Cell migration was studied on LLC and MDA-MB-231 cells using the scratch assay. MDA-MB-231 presented a dose dependent reduction in cell migration. Under the same concentrations, LLC completely detached from the surface and, therefore, we reduced the doses in these cells. Even after x40 dilution of the drug, similar effects were observed (Figure 1, Supplementary Figure 3). This suggests that cells from different origins possess varying adherence potentials, allowing the drug to provoke a reaction that is correlated with cell susceptibility.

Transwell assay is an *in vitro* 3D based assay for testing cell migration. In this model, tumor cells invade through a porous membrane and are then stained and counted. Our results show significant decrease in migration of LLC cells over 21 hours (Figure 2). Combined, these results prove that Carbenoxolone can inhibit cell migration using low, non-toxic concentrations, and suggest LLC's ability of adhesion and colony formation to be particularly sensitive to Carbenoxolone, a hypothesis which was later confirmed.

Once in the circulation, cells lose their contact with the ECM and are susceptible to a programmed cell death known as anoikis. While survival rates of these epithelial cells under these conditions are rather limited, those which do evade anoikis continue the metastatic process [40]. Increasing susceptibility of cancer cells to anoikis due to Carbenoxolone treatment was reported in the past in the human thyroid cancer cell line treated with high concentrations (up to 50 μM), and was attributed to the loss of gap junctions [29]. We show that in LLC cells, substantially lower doses of Carbenoxolone, as low as 0.1 μM, can already enhance cell susceptibility to anoikis.

To disseminate, tumor cells utilize a central mechanism in the metastatic process, namely, the molecule-dependent epithelial cell adhesion [41]. Cell adhesion to both the endothelial layer and tissues of the targeted organs is a central mechanism in tumor metastases [42]. Several endothelial adhesion molecules, such as E-selectin [43, 44] and E-cadherin, have been identified to mediate metastatic cell facilitation, thus further emphasizing the importance of investigating the role of adhesion in the formation of metastasis in the lung [20, 43, 44].

Both adherence to ECM and potential of adherence to endothelium via adhesion molecules were assessed. By coating culture plates with various components of the ECM: elastin, fibronectin, collagen, gelatin and laminin, we aimed to investigate the effect of Carbenoxolone on cell adhesion and to provide an initial glimpse into the cellular mechanisms involved in metastases to the lung. Data show (Figure 2, Supplementary Figure 5 and Supplementary Figure 7) that cells incubated in collagen-coated wells significantly lost their adhesion capability under treatment in a dose-dependent manner. These data suggest that adhesion molecules which bind to collagen are affected by the drug. However, we could not detect modifications in α1 and α2 integrin mediated adhesion (data not shown). In addition to the effect of Carbenoxolone on adhesion to collagen, our results show that the drug dramatically affected the capacity of LLC cells to form colonies in soft agar (Figure 2B). Cell organization and colony formation *in vitro* requires cell migration, cell–cell interactions and proliferation. Since the soft-agar assay is made with pre-seeded cells in agar matrix, cell adherence to ECM is less relevant in this assay. We found only minor anti-proliferation effects of the drug, thus we can determine that the primary effect of the drug in this assay is via migration and cell–cell interaction rather than proliferation or adhesion. The ability to attach to lung endothelium and the capacity of cancer cells to adhere to each other to form colonies are key processes in lung metastasis which are affected by the drug. ICAM1 is among the key adhesion molecules known to play an important role in cancer metastases [45–48], possibly through colonization [46], and was suggested to enhance tumor growth being a pro-angiogenic factor [43, 47, 48]. Considering our observations, we suggest a possible molecular mechanism explaining both the *in vitro* colony formation experiment and the *in vivo* tail vein model results.

Immunostaining of LLC cells treated with Carbenoxolone presented lower levels of both cellular HMGB1 and membrane ICAM1. Results were further confirmed by immunoblot analysis and flow cytometry. Based on these findings, we investigated ICAM1 protein localization and expression in tumor tissue sections from the subcutaneous (s.c.) experiment, which confirmed reduced levels of both proteins *in situ* (Figure 6). Carbenoxolone mediation of ICAM1 may be attributed to HMGB1 signaling pathway. HMGB1 activates nuclear factor kappa b (NF-κB) via RAGE, which, in turn, induces the pro-inflammatory mediator tumor necrosis factor alpha (TNF-α) [49] that upregulates ICAM1 [44]. In further support of our findings, GA was shown to reduce ICAM1 expression in TNF-α-stimulated HUVEC cells through the blockade of NF-κB [50].

Carbenoxolone is known to modify gap junctions via connexin43 [30, 39, 51]. Connexin43 is a protein that mediates both intracellular and extracellular communications, and its effect on tumor progression remains inconclusive with studies presenting both suppression [51, 52] and induction [29, 30, 39] of growth in different cancer cell lines. Yet, studies suggest a gap junction independent anti-cancer mechanism, as indicated by preserved anti-cancer traits when cells were treated with Carbenoxolone [52].

To validate that the activity of Carbenoxolone is maintained *in vivo*, we used both primary and secondary tumor models. S.c. model in C57BL/6J mice treated by 50 mg/kg of Carbenoxolone i.p. q.o.d, showed no significant reduction in tumor growth and volume. However, notably, despite the similar volume, immunoblotting and immunohistology staining indicated lower levels of HMGB1 and vascularization in tumor tissue, suggesting a regulation of a tumor niche (Figure 3D, Supplementary Figure 6). These effects on tissue niches can potentially be valid also in distant organs affecting the colonization of tumor cells. However, since the s.c. model is limited in the sense that cells do not grow in their physiological microenvironment, we used a recently developed orthotopic model of primary lung cancer, providing a better model for mimicking the progression of primary lung cancer and its pathological behavior in its relevant microenvironment [16]. Yet, histological analysis of the lungs (Figure 4A) showed no effect on primary tumor growth, further supporting our s.c. data and our hypothesis that the effect of the drug is mainly on the metastatic cells rather than the primary tumor.

The *in vivo* model of tail vein injection is a method commonly used to investigate the anti-metastatic effect of drugs [53]. Although this model lacks the spontaneous dissemination of tumor cells, it can indicate the ability of tumor cells to survive in the circulation and colonize in the lung tissue. Indeed, lungs removed from mice treated with Carbenoxolone after 26 days, showed a significant reduction in the number of lesions detected in lung tissues, and the size of the detected lesions was smaller compared with the untreated group. Results correlate with the significant difference of lung weight, which can be attributed to metastases and edema (Figure 4B-4E). Our *in vivo* data are in line with the *in vitro* observation which shows reduction in cell adhesion to collagen and colony formation in soft agar. Similar results were reported for Isoliquiritigenin, a Licorice extract compound, which demonstrated the reduction of lung lesions in murine renal carcinoma using the tail vein injection model [54].

**Figure 7:**
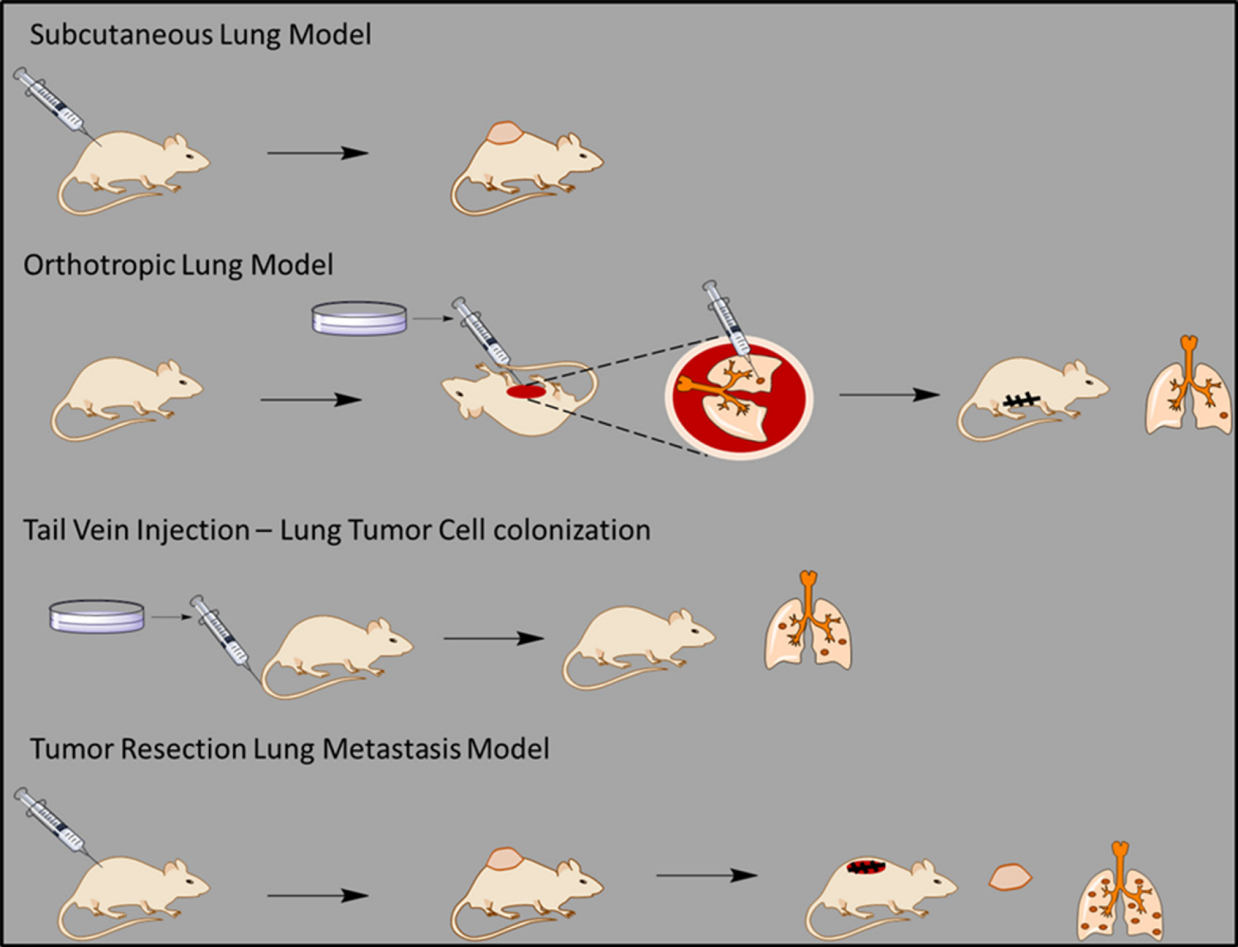
A general scheme of all the *in vivo* experiments A scheme describing the different *in vivo* models used in our study on the effect of Carbenoxolone on lung metastasis.

Since the tail vein injection model detects mostly the ability of cells to colonize in the lungs, we followed up our investigation using tumor resection model which involves intravasation, anoikis, adhesion, colonization and migration (Figure 5, Figure 7, Table 1). In this model, we found that at the end point of the experiment, the number of metastatic lesions in the lungs of Carbenoxolone treated mice were substantially lower than that found on the lungs of the untreated mice. While the control group presented significantly more lesions, the higher weight of their resected lungs may be partially attributed to edema and metastases.

**Table 1:** An overall summary of the different treatments and results of all *in vivo* models that were applied in our study on the effect of Carbenoxolone on lung metastasis

Model	Relevant processes in cancer formation	Dosage	Duration	Results
Subcutaneous Lung Model	Proliferation	50 mg/kg every other day	17 days	No significant difference
Orthotropic Lung Model	Proliferation	10 mg/kg every day	17 days	No significant difference
Tail Vein Injection – Lung Tumor Cell colonization	Adhesion Anoikis Colonization	40 mg/kg every other day	25 days	Treated group presented significantly lower number of lesions on lungs (*p* = 0.006)
Tumor Resection Lung Metastasis Model	Adhesion Anoikis Colonization Intravasation Migration	50 mg/kg every other day	38 days	Treated group presented significantly lower number of lesions on lungs (*p* = 0.002)

In conclusion, we have demonstrated that Carbenoxolone has a clear anti-metastatic effect, mediated mainly by the reduction of cell interactions via downregulation of ICAM1 and possibly the suppression of HMGB1 in the lung. These results suggest the potential use of Carbenoxolone, a non-toxic, approved and prescribed drug, as a prophylactic treatment for metastasis formation in the lung following the diagnosis of a primary tumor.

## MATERIALS AND METHODS

Unless specified otherwise, incubations of Carbenoxolone were performed using 0, 0.1, 1 and 3 μM over 1 hour. Carbenoxolone was purchased from Sigma-Aldrich (CAS number: 7421401)

### Viability assay

Each well of a 96-well culture plate was seeded with 4000 cells of; NIH/3T3, A-375 and LLC, in 100 μl media (DMEM, 10% FCS) and incubated in 37°C for 24 hours. A total of three plates with each cell line seeded in a separate plate. Treatment of Carbenoxolone in varying concentrations (0, 0.1, 0.5, 1, 5 and 10 μM) was added in another 100 μl media and cells were incubated in 37°C for another 72 hours. After the incubation, WST-8 reagent was added into each well and cells were incubated in 37°C for 1 hour. Absorbance was measured at 450 nm using a plate reader (Wallac 1420 VICTOR plate-reader, Perkin-Elmer Life Sciences, USA).

### Macrophage activation assay

RAW 264.7 cells (4 × 106 cells/ml) were plated in 6-well plates and subsequently treated with or without LPS (1 μg/ml) in the presence of different concentrations of Carbenoxolone: 0, 10, 50, and 100 μM, for 24 h. Ethylene pyruvate-1 (EPI-1) was added as positive control. Cell lysates were prepared (described below) and production of HMGB1 was assessed using Western blot analysis.

### Transwell based migration assay

LLC cells were harvested and centrifuged for 5 minutes, at room temperature (RT). Next, cells were suspended in serum free media and counted. For this assay we used a 24-well cultured plate with 8 μm pore size polycarbonate membrane transwell inserts (NuNC). 1 ml of full media (10% FCS) was added to the lower compartment, and all inserts were adjusted so that the membrane would be submerged in media. The drug was applied in the growth media for 1 hour incubation. Next, 200 μl of the 2.5 × 10^5^ /ml cell suspension (DMEM, 0% FCS) were added to each of the insert's upper compartments. Cells were then incubated in 37°C for 21 hours to allow cells to migrate toward the underside of the insert's filter. Media was removed from lower compartment and cells fixed on the lower side of the insert's filter quickly with 4% paraformaldehyde (PFA) for 5 minutes and washed twice with PBS. We then incubated cells with 80% Methanol for 20 minutes and washed twice with PBS. Staining of the cells on the lower side of the insert's filter was done using Giemsa stain for 20 minutes at RT protected from light. After 20 minutes we carefully swabbed the upper side of the membrane. Staining buffer was removed and inserts air-dried. Using a microscope, cells on the lower side of the insert's filter were counted, three different fields per well, and the average number of migrated cells was calculated.

### Scratch assay

Scratch inserts (ibidi, Catalog number: 80209) were attached onto the bottom of 24-well culture plate wells. LLC cells and MDA-MB-231 cells were harvested and centrifuged for 5 minutes at RT. Cells suspended in media and counted to get 6 × 10^5^ cells/ml. 70 μl of cell stock was applied in each well. Cells were then incubated in 37°C for 24 hours to allow appropriate attachment, after which media discarded and treatments were applied proceeded by a 1 hour incubation in 37°C. Different concentrations of Carbenoxolone were added for each cell line: LLC- 0, 0.025, 0.05 and 0.1 μM and MDA-MB-231. Culture inserts were removed using sterile tweezers and cells were washed carefully with PBS to remove cell debris. Serum free media (DMEM, 0% FCS) was added into each well and pictures were taken using a microscope (t 0), after which cells were incubated in 37°C and pictures were taken again after 8 hours (t 8) and 16 hours (t 16).

### Anoikis assay

Anoikis was induced using p-HEMA culture. A solution containing 20 mg/ml p-HEMA in 95% ethanol was made and left in RT to dissolve. Once dissolved, solution was pipetted into 6 well culture plates. The plates were left half covered in sterile environment on a rocking plate, until the ethanol evaporated and the p-HEMA solidified and coated plated evenly. Plates were then washed twice with PBS to remove possible traces of ethanol. Each plate was incubated with growth media containing different concentrations of Carbenoxolone. All plates were seeded with 50,000 LLC cells/well and incubated for 72 hours in 37°C. Cell viability was measured using WST8 as described above.

### Soft agar assay

To study the effect of Carbenoxolone on the potential of LLC to form colonies, soft agar assay was performed [55]. Each well of a six-well culture plate was coated with 2 ml bottom agar mixture (DMEM, 10% FCS, 2% agar). After the bottom layer had solidified, 1 ml top agar mixture (DMEM, 10% FCS, 0.6% agar) containing 7,500 LLC cells per well was added. After this layer had solidified, wells were overlaid in an additional 1 ml of full media with different concentrations of Carbenoxolone: 0, 0.1, 1, and 3 μM. Plates were incubated at 37°C and media with treatment was refreshed every 2 days. On day 12, colonies were visualized using a light microscope and colonies from different fields of view were counted and photographed under ×10 magnification. The average number of colonies per well was calculated. Next, wells were incubated with Giemsa in RT for 2 hours and then photographed.

### Adhesion assay

The effect of ECM on Carbenoxolone-mediated cell adhesion, was studied using five different coatings with ECM component. Each well of a 24-well culture plate was coated with 0.5 ml of: elastin, fibronectin, collagen (I), gelatin and laminin (50 μg/ml) and one plate was left uncoated for control. Coatings were left 2 hours at RT. Plates were washed with 1 ml PBS and incubated with media for 30 minutes in 37°C. LLC cells were harvested and counted. 2 × 10^6^ cells were suspended in 2 ml PBS, 10 μl DiO were added with the tube kept on ice under aluminum foil for 20 min. 6.7 × 10^4^ were seeded in each well and incubated in 37°C, under treatment of Carbenoxolone for 1 hour. Cells were washed twice with PBS and total fluorescent signal was measured (at the central field) using a plate reader (Wallac 1420 VICTOR plate-reader, Perkin-Elmer Life Sciences, USA) at Ex/Em of 480/530.

### Subcutaneous model

Eight week old C57BL/6J mice (Harlen, Israel) were inoculated s.c. with 1.5 × 10^6^ LLC cells. When tumors reached ~100 mm^3^, animals were divided into two groups and treatment was administered as 50 mg/kg q.o.d (i.p.) or PBS as control. The tumor growth was measured transcutaneously with a caliper every other day until tumors reached a volume of ~1000 mm^3^. Tumor volume was calculated and expressed as a mean ± SE. At the end point (day 17), mice were sacrificed and tumors were harvested. Tumors were measured and weighed before embedded in an optimum cutting temperature compound (OCT), frozen on dry ice, and stored at −80°C. Frozen sections (12 μm) were cut using a cryostat (−20/−19°C).

### Immunofluorescence staining

All incubations were performed at RT and all reagents were rinsed with 1× PBS. Sections were fixed in 4% PFA for 20 minutes and washed 3 times for 5 minutes. Triton 0.1% incubation of 20 minutes was used for permeability followed by 3 washes as earlier described. Sections were incubated in 3% normal goat serum for blocking. Blocking was removed and anti-ICAM1 (abcam 1:200) or anti-HMGB1 (abcam 1:250) or anti-CD31 (abcam 1:50) in 3% normal horse serum was added for overnight incubation in 4°C. Slides were washed twice for 5 minutes and incubated with a secondary antibody labeled with Cy3 (mouse anti-rabbit, abcam) (1:500) in 3% normal goat serum for 1 hour and washed twice before fluorescent mounting with DAPI (VestaShield, Vector laboratories) applied on the slides, and samples were visualized using a fluorescent microscope (Olympus EX-73).

### Preparation of tumor lysates

Upon harvesting the tumors of the s.c. experiment, portions of approximately 3 mm^2^ were taken of each tumor and stored at −80°C. Lysates were made using the Bullet Blender^®^ tissue homogenizer (ISS) with 0.5mm zirconium oxide beads (ZROB05). RIPA was added to each sample (300 μl) o just so to cover the tumor, along with 1 spoon of beads. Homogenizer was set to speed 10 and activated for 2 minutes. Samples were then centrifuged in a cold centrifuge (4°C) for 10 minutes at 12.3 rpm. Soup was collected and samples were kept on ice. BCA was used to determine protein level as further detailed and 25 μg of protein was taken for western blot analysis.

### Orthotopic model

The experiment was performed based on the method previously published [16]. 14 C57BL/6J mice were injected with ~700 LLC cells/mouse into the lungs. 3 days following tumor cell implantation, 7 mice were injected with Carbenoxolone 10 mg/kg per day (i.p.). Treatment was given over 14 days with the other 7 mice serving as the control group. Mice were then sacrificed and left lung was sent for histological serial section analysis.

### Tail vein

Eight weeks old C57BL/6J mice (Harlen, Israel) were pretreated with 40 mg/kg Carbenoxolone or PBS for control (8 mice) every other day. On the 5th day, all mice were injected intravenously (i.v.) with 5 × 10^6^ LLC cells in 100 μl PBS. 21 days after treatment started, 1 mouse of the control group died and all mice were then sacrificed. Lungs were harvested, weighed and left in 4% formalin overnight. Lungs were then transferred to 85% ethanol and sent for histological serial section analysis. Lesion on lungs were counted using the hematoxylin and eosin stained sections. Lesions were divided into 3 groups: Small, medium and large, and after counting how many of each group were found on the slide, the total number of lesions per slide was calculated (Figure 4D).

### Resection model

Fourteen eight weeks C57BL/6J mice were inoculated s.c. with 1.5 × 10^6^ LLC cells. Dose was set to be 50 mg/kg q.o.d in i.p, which is the maximum tolerated dose (MTD) of Carbenoxolone, as found in our preliminary experiments showing that 60 mg/kg q.o.d led to over 10% weigh loss. Treatment of Carbenoxolone 50 mg/kg i.p. 3 times a week was administered 1 week prior to injection of cells, while the control group was left untreated. After 10 days, when tumors reached volume of ~1000 mm^3^, mice were anesthetized and tumors were resected surgically. Mice were monitored for an additional 21 days to allow metastases to develop. At the end point (day 38), mice were sacrificed and lungs were harvested and weighed and lesions on the surface of the lungs were counted.

### Immunofluorescence staining of cultured cells

LLC cells were harvested and counted. 5 × 10^4^ cells were seeded on glass cover slips (Ø 22 mm) placed in wells of a sterile 24-well culture plate with 1 ml media, and incubated overnight in 37°C for proper attachment. Media was removed and cells washed twice with PBS. Drug was applied in the growth media and cells were incubated in 37°C for 1 hour. Cells were then carefully washed twice with PBS and fixed with 4% PFA for 20 minutes in RT followed by three washes of PBS. For tissue permeability, 0.1% Triton was added for a 10 minutes RT incubation followed by blocking with 2% BSA (bovine serum albumin) in PBS for 30 minutes RT. Anti-ICAM1 (abcam, 1:200) or anti-HMGB1 (abcam, 1:400) in 2% BSA was added for overnight incubation in 4°C. Cells were washed three times with PBS, proceeded by the addition of either Alexa fluor 488 (mouse anti-rabbit, abcam) (for HMGB1 detection, or Cy3 conjugated secondary antibody (mouse anti-rabbit, abcam) (1:50) for ICAM1 detection, in 2% BSA for 1 hour incubation in RT, in dark. Cells were washed twice with PBS, mounted with fluorescent mounting with DAPI (VestaShield, Vector laboratories) and applied on slides visualized using a fluorescent microscope (Olympus EX-73).

### Immunoblotting

LLC cells were harvested and counted and cells were incubated overnight to ensure proper attachment. Carbenoxolone was added to the media and cells were incubated for 1 hour in 37°C after which they were harvested and centrifuged. Pellet was washed twice with PBS and suspended in RIPA buffer (20 mM Tris·HCl pH 7.5, 140 mM NaCl, 1% NP-40, 1mM sodium orthovanadate, 0.1% SDS) with protease inhibitors for 30 minutes on ice followed by vigorous pipettation. Cell lysate was centrifuged for 10 minutes at 1.3 g in 4°C and pellet was discarded. Protein concentration measured using a BCA protein assay kit (Pierce, Catalog number: 23227). Samples (30 μg protein) were resolved by SDS-PAGE (12% acrylamide) and transferred to PVDF (Polyvinylidene fluoride) membranes. Blots were incubated with primary antibodies anti-HMGB1 and anti-ICAM1 overnight at 4°C. Anti-rabbit HRP-conjugated secondary antibody (abcam, Catalog number: 49900) was used followed by chemiluminescence detection. Acquisition was done using Bio-Rad Molecular Imager®.

### Flow cytometry

The effect of Carbenoxolone on ICAM1 levels was studied using the flow-cytometry method. LLC cells were harvested and counted and cells were seeded into 6 well culture plates, cells were incubated overnight to ensure proper attachment. Carbenoxolone (0, 0.1, 0.25, 0.5 and 1 μM) was added to the media and cells were incubated for 1 hour in 37°C after which they were harvested and centrifuged. Pellet was washed twice with cold PBS and stained with ICAM1 (abcam, Catalog number: 124760, 2 μg/ml) in RT for 1 hour. Cells were washed 3 times with PBS and centrifuged at 1.3 rpm in 4°C after which they were stained with Cy3 (mouse anti-rabbit, abcam) (1:200) for 30 min. PBS was used for washing and cells were suspended in FACS (Fluorescence Activation Cell Sorting) buffer (0.05% NaNO_3_, 1% BSA) and analyzed using a FACS machine (CytoFLEX, Beckman Coulter).

### Statistics

*In vitro* data is presented as mean ± SD whereas *in vivo* data is presented as mean ± SE. Significant differences between cell viability, migration, number of colonies or tumors, tumor volume and tumor weight, were assessed using unpaired two-tailed Student's *t-test*, and *p* < 0.05 was considered statistically significant.

### Cell culture

All cell lines were characterized and purchased from ATCC. Cells were used for experiment up to p20 and were Mycoplasma free, using EZ-PCR Mycoplasma Test Kit (Biological Industries, Catalog number: 2070020).

### Animal studies

All institutional and national guidelines for the care and use of laboratory animals were followed (protocols: MD-14-14054-5, MD-16-14648-5 and MD-14-14021-5)

### Ethics approval

All institutional and national guidelines for the care and use of laboratory animals were followed and protocols were approved by the Hebrew University Ein Karem Medical School IACUC (protocols: MD-14-14054-5, MD-16-14648-5 and MD-14-14021-5)

## SUPPLEMENTARY MATERIALS FIGURES



## Abbreviations

RAGE: Receptor for Advanced Glycation End products
HMGB1: High Mobility Group Box 1
ICAM1: Intercellular Adhesion Molecule 1
LLC: Lewis Lung Carcinoma
ECM: Extra Cellular Matrix
LPS: Lipopolysaccharide
EPI-1: Ethylene pyruvate-1
PVDF: Polyvinylidene Fluoride
S.C.: Subcutaneously
I.P.: Intraperitoneally
I.V.: Intravenously
GA: Glycyrrhetinic Acid
NF-κB: Nuclear factor kappa b
TNF-α: Tumor necrosis factor alpha
