# Supplementary material for: High mobility group box 1 antagonist limits metastatic seeding in the lungs via reduction of cell–cell adhesion
Source: Oncotarget. 2017 Mar 14;8(20):32706–21. doi: 10.18632/oncotarget.16188 (PMC5464821; doi:10.18632/oncotarget.16188)
Supplement: Supplementary file 1 [file oncotarget-08-32706-s001.pdf]

High mobility group box 1 antagonist limits metastatic seeding in the lungs via reduction of cell-cell adhesion

Supplementary Materials

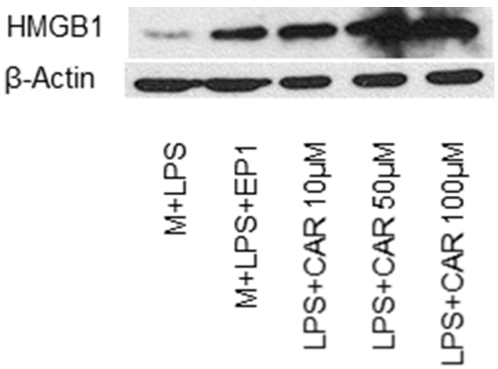

Supplementary Figure 1: Carbenoxolone inhibits LPS-induced HMGB1 secretion: Western blot analysis of Carbenoxolone activation using LPS activation assay. LPS activated Macrophages (M) were incubated with EPI-1 as positive control.

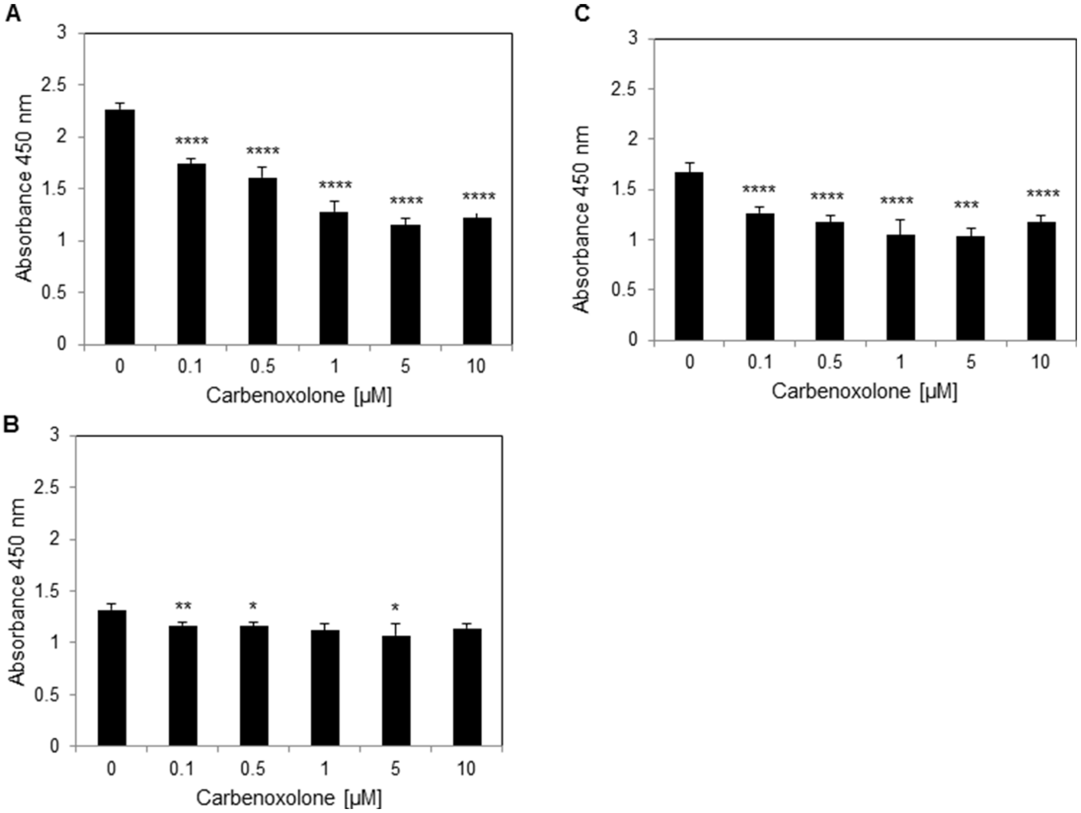

Supplementary Figure 2: Carbenoxolone affects proliferation in different cell lines. Cell proliferation of (**A**) A-375 (**B**) LLC and (**C**) NIH/3T3 cell lines after 72 hours of incubation with Carbenoxolone. *n* = 12. *$p \leq 0.05$, **$p \leq 0.01$, ***$p \leq 0.001$, ****$p \leq 0.0001$.

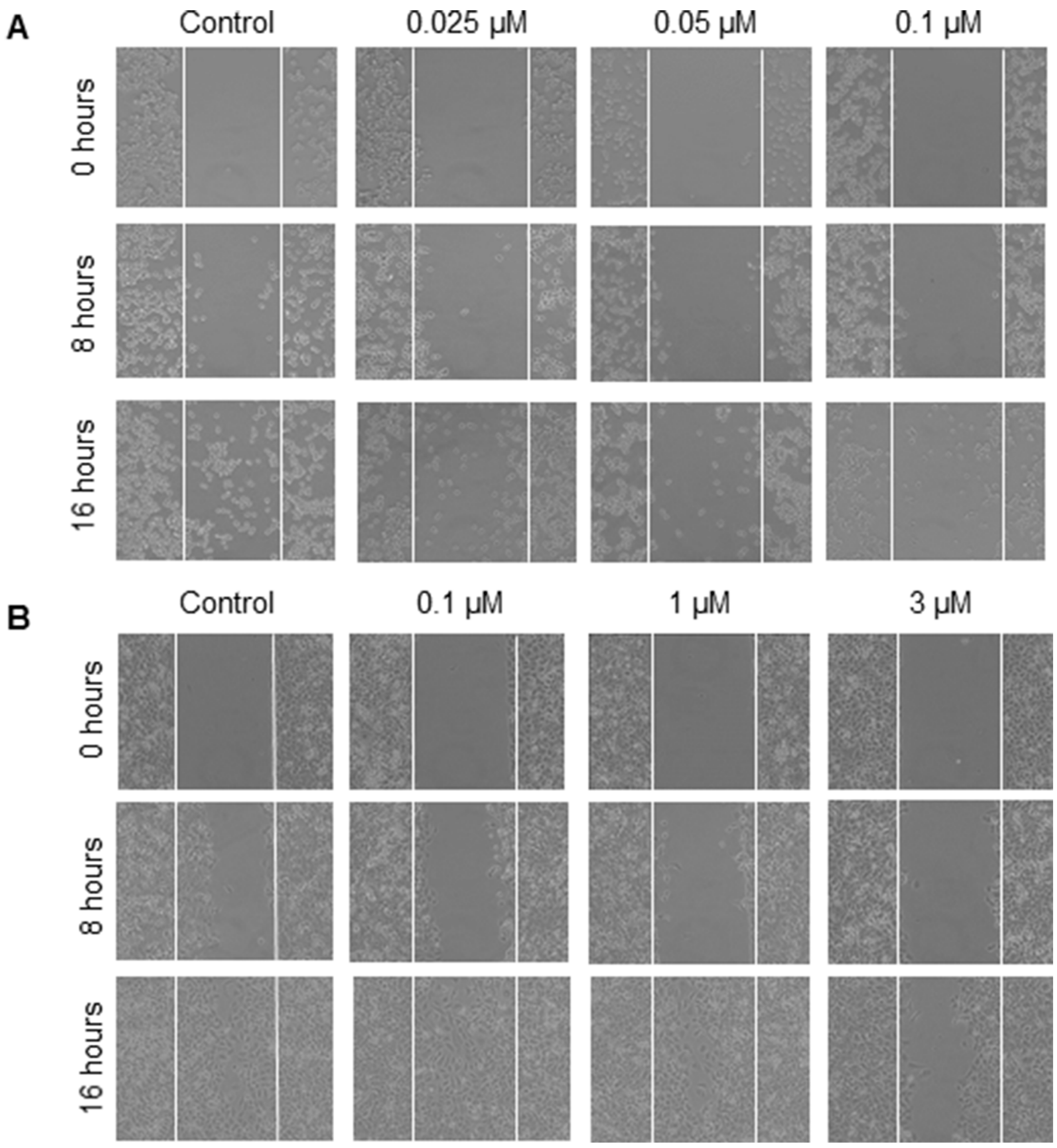

Supplementary Figure 3: Effect of Carbenoxolone on cell migration. Wound healing of (**A**) LLC and (**B**) MDA-MB-231 at t0 and post 8 and 16 hours of incubation with Carbenoxolone, with different drug concentration. White thin lines represent the scratch area. *n* = 3.

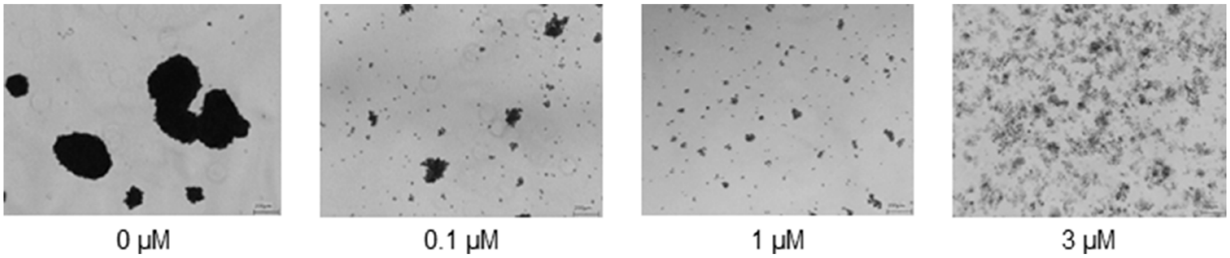

Supplementary Figure 4: Carbenoxolone increases cells' susceptibility to anoikis. Anoikis assay using LLC incubated for 72 hours Carbenoxolone. 100K cells were seeded on p-HEMA coated plates, and cell viability was measured using WST8 with absorbance detected at 450nm using a plate reader. *n* = 6.

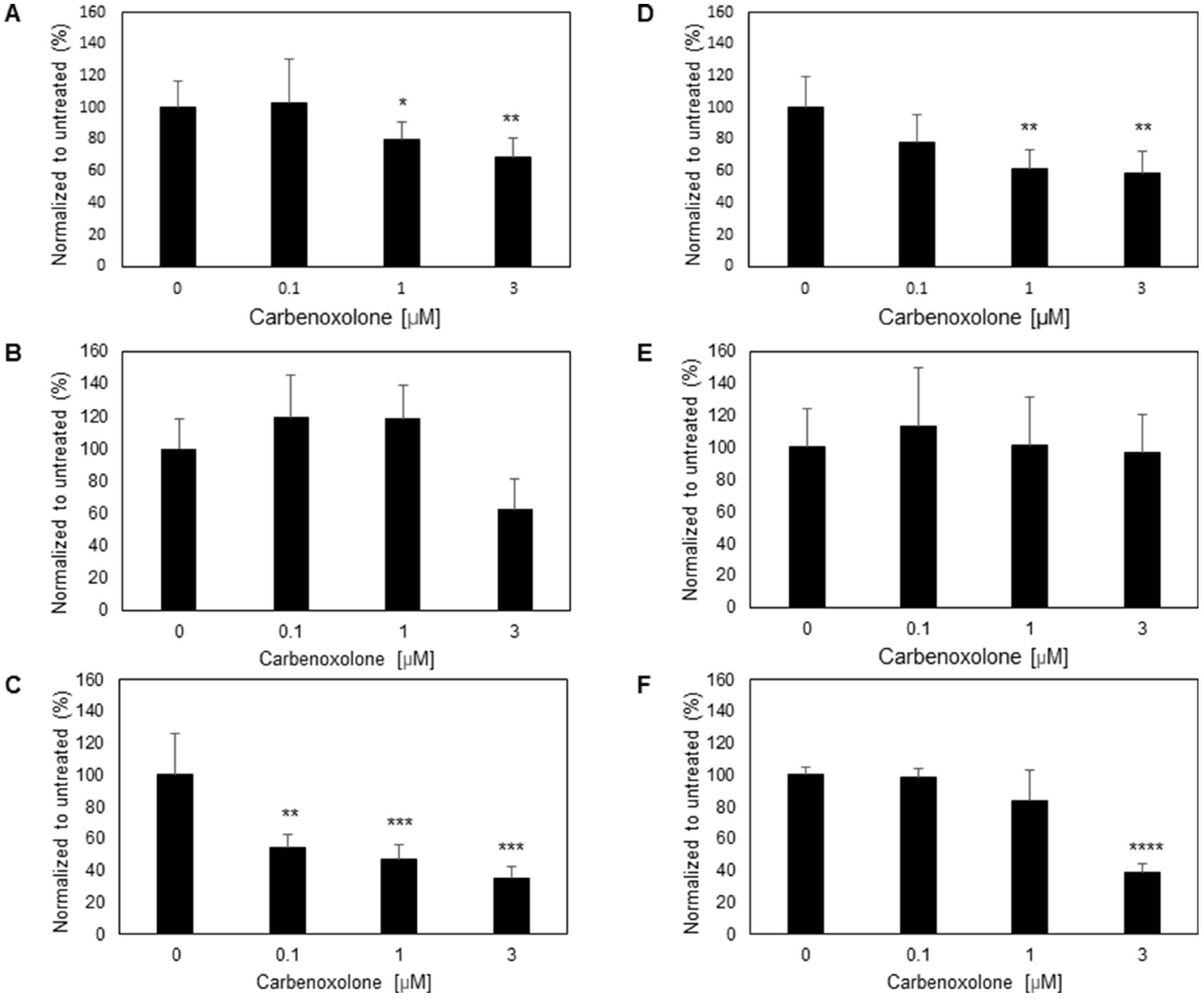

Supplementary Figure 5: Collagen is the most affected ECM component resulting Carbenoxolone treatment. Adhesion assay of LLC on plates coated with varying extra cellular matrix components post incubated with Carbenoxolone for 1 hour. Results were normalized compared to the untreated cells of the (**A**) uncoated plate. Coatings were of (**B**) fibronectin (**C**) collagen (**D**) Laminin (**E**) Elastin and (**F**) Gelatin. $n = 6$. *$p \leq 0.05$, **$p \leq 0.01$, ***$p \leq 0.001$, ****$p \leq 0.0001$.

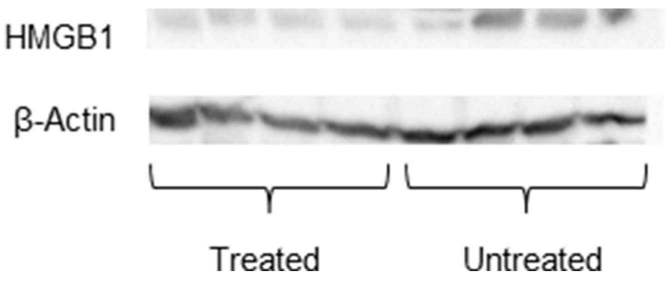

Supplementary Figure 6: Carbenoxolone lowers level of HMGB1 in tumors of treated mice. Level of HMGB1 in tumors of either treated by Carbenoxolone or untreated mice. *n* = 4.

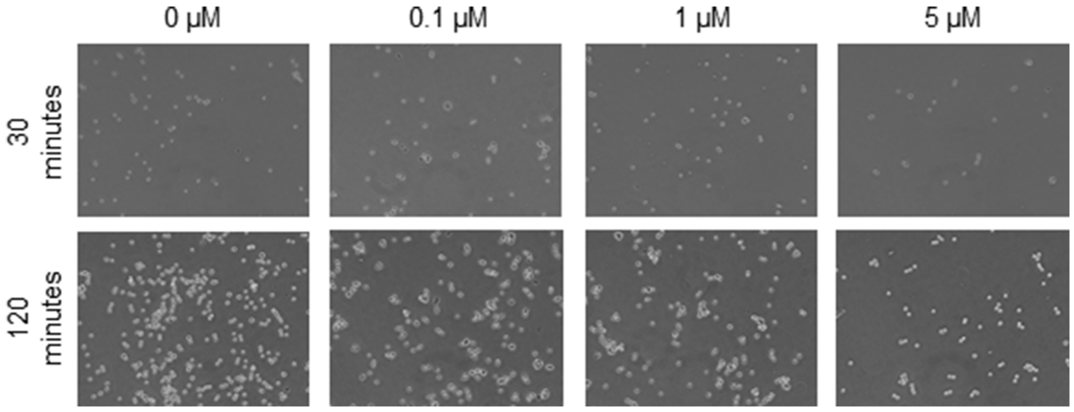

Supplementary Figure 7: Carbenoxolone has a concentration dependent effect on LLC cells. Adhesion of LLC cells on uncoated culture plates, post 30 and 120 min of incubation with Carbenoxolone. *n* = 6.
